# Unveiling the dynamic of nitrogen through migration and transformation patterns in the groundwater level fluctuation zone of a different hyporheic zone sediment

**DOI:** 10.1038/s41598-024-54571-2

**Published:** 2024-02-17

**Authors:** Yusuf Abdulhamid, Lei Duan, Sun Yaqiao, Jinmei Hu

**Affiliations:** 1 School of Water and Environment, Chang’an University, Xi’an, 710054 China; 2 Key Laboratory of Subsurface Hydrology and Ecological Effects in Arid Region, Ministry of Education, Chang’an University, Xi’an, 710054 China; 3 Department of Plant Science and Biotechnology, Federal University, PMB 5001, Dutsin-Ma, Katsina State Nigeria

**Keywords:** Nitrogen dynamics, Hyporheic zone soil, Groundwater levels, Soil texture, Biogeochemical processes, Biogeochemistry, Environmental sciences

## Abstract

This study investigates the impact of water levels and soil texture on the migration and transformation of nitrate (NO_3_^−^-N) and ammonium (NH_4_^+^-N) within a soil column. The concentrations of NO_3_^−^-N gradually decreased from an initial concentration of 34.19 ± 0.86 mg/L to 14.33 ± 0.77 mg/L on day 70, exhibiting fluctuations and migration influenced by water levels and soil texture. Higher water levels were associated with decreased NO_3_^−^-N concentrations, while lower water levels resulted in increased concentrations. The retention and absorption capacity for NO_3_^−^-N were highest in fine sand soil, followed by medium sand and coarse sand, highlighting the significance of soil texture in nitrate movement and retention. The analysis of variance (ANOVA) confirmed statistically significant variations in pH, dissolve oxygen and oxidation–reduction potential across the soil columns (p < 0.05). Fluctuating water levels influenced the migration and transformation of NO_3_^−^-N, with distinct patterns observed in different soil textures. Water level fluctuations also impacted the migration and transformation of NH_4_^+^-N, with higher water levels associated with increased concentrations and lower water levels resulting in decreased concentrations. Among the soil types considered, medium sand exhibited the highest absorption capacity for NH_4_^+^-N. These findings underscore the significant roles of water levels, soil texture, and soil type in the migration, transformation, and absorption of nitrogen compounds within soil columns. The results contribute to a better understanding of nitrogen dynamics under varying water levels and environmental conditions, providing valuable insights into the patterns of nitrogen migration and transformation in small-scale soil column experiments.

## Introduction

Nitrogen (N) is an essential element for plant growth and ecosystem functioning^1^. However, excessive N inputs from human activities, such as agriculture and industrial emissions, have resulted in significant environmental concerns. N pollution contributes to water eutrophication, soil degradation, air pollution, and adverse effects on human health and biodiversity^2,3^. Understanding the movement and transformation of N in soil systems is crucial for managing N pollution, optimizing nutrient retention, and promoting sustainable agricultural and natural environments^4,5^. The migration and transformation processes significantly influence the fate of N compounds in soil. Various forms of N, such as NO_3_^−^-N, ammonium nitrogen (NH_4_^+^-N), and nitrite nitrogen (NO_2_^−^-N), undergo transformation reactions driven by microbial activity, soil processes, and environmental conditions^4,6^.

Moreover, the movement of N in soil is influenced by factors like water levels, soil texture, porosity, and hydraulic properties^7–10^. Water levels, in particular, have a profound impact on N compound migration and transformation, affecting oxygen availability, microbial activities, and nitrification and denitrification processes^1,11^. Additionally, soil texture, characterized by particle size distribution, influences water-holding capacity, nutrient retention, and permeability^12,13^. Different soil textures exhibit varying absorption capacities for N compounds, consequently affecting their movement and retention in the soil^14^. Research on N migration and transformation in soil columns provides valuable insights into the complex dynamics of N compounds under different water levels and soil textures^15^. By monitoring the concentrations of NO_3_^−^N, NH_4_^+^-N, and NO_2_^−^-N at various depths within soil columns over a designated time period, researchers can investigate the response of N compounds to changing environmental conditions^16^. Previous studies have contributed to our understanding of the factors controlling N migration and transformation, providing insights for sustainable soil management and N pollution mitigation strategies^17,18^. In recent years, there has been a focus on studying the migration of pollutants, such as non-aqueous phase liquids (NAPL) and heavy metals, in porous media under groundwater level fluctuations^19–21^. Several studies have been conducted to investigate the migration of benzene series pollutants and crude oil in heterogeneous soil layers using indoor soil column experiments and monitoring wells. Groundwater level fluctuations have been found to impact the concentration of arsenic in groundwater in the Hanjiang Plain. The effect of water level fluctuations on the migration and transformation of iron has also been studied using a sand column experiment^22–25^. However, there has been relatively little research conducted on the migration of nitrogen under water level fluctuations.

Some studies have shown that water level fluctuations can promote the migration of NO_3_^−^N, leading to increased nitrate pollution in groundwater^26^. The content of NO_3_^−^N has been found to increase with nitrogen application and water level fluctuations, indicating a joint effect on the concentration of nitrate-nitrogen^27,28^. Another study demonstrated that shallow groundwater leaching can increase the content of nitrate-nitrogen and ammonium nitrogen in groundwater, with the depth of groundwater burial influencing the nitrogen content^29^. While there have been studies on the mechanism of nitrogen migration and transformation, research on the effects of groundwater level fluctuation on nitrogen migration and transformation is still limited. Existing studies have mainly focused on a single soil medium, which fails to capture the differences in nitrogen migration and transformation between different media under water level fluctuations. Additionally, the pollutants considered have primarily been NO_3_^−^N. However, there is a need for more systematic research on the mechanisms of nitrogen migration and transformation under groundwater level fluctuations. This research should consider different media and investigate additional pollutants beyond nitrate nitrogen. This study explore the migration and transformation of N compounds (NO_3_^−^-N, NH_4_^+^-N, and NO_2_^−^-N in soil columns under varying water levels and soil textures. Based on existing knowledge and observations, it is hypothesized that water levels and soil texture significantly influence the migration, transformation, and absorption of N compounds in soil systems. Changes in water levels are anticipated to impact the availability of oxygen, nitrification and denitrification processes, consequently affecting the concentrations of NO_3_^−^-N, NH_4_^+^-N, and NO_2_^−^-N. Furthermore, different soil textures are expected to exhibit varying absorption capacities for N compounds, leading to differences in their movement and retention in the soil.

The specific objectives of this study are as follows: (1) Investigate the impact of water levels on the migration and transformation of NO_3_^−^-N, NH_4_^+^-N, and NO_2_^−^-N in soil columns; (2) Assess the absorption capacities of different soil textures (coarse sand, medium sand, and fine sand) for N compounds; (3) Analyze the temporal variations in N concentrations at different depths within soil columns and elucidate the influencing factors; and (4) Examine the sequence of N absorption rates for each soil texture under varying water levels. By addressing these objectives, The study aimed to unravel the intricate pattern of N movement and absorption in soil systems and contribute to our understanding of biogeochemical processes related to N dynamics but limited to the relationship between the N and other variables or sediment.

The findings of this study will provide a scientific basis for the remediation and treatment of soil, groundwater pollution as well as promoting sustainable soil and wastewater management practices. The results may also attract the attention of researchers, environmentalists, and agricultural professionals interested in understanding and addressing N pollution and its impacts on the environment.

## Materials and methods

### Sample collection and testing

The study area selected for this research is the Weinan section of the Wei River Basin, which is the largest tributary of the Yellow River and serves as an important economic core area and ecological corridor in the Loess Plateau. The Weihe river is situated next to the Loess Plateau in the northern region and the North Qinling Mountains in the southern region. In Shaanxi, the main stream of the river spans a length of 502.4 km, with a drainage area covering 67,108 km^2^^30^. The Weihe River basin encompasses a wide range of landscapes, climate patterns, and human activities, extending from its upper floodplain in Yangling to the lower reaches of Weinan. The geomorphological profile of the Weihe River basin is diverse. The upper floodplain in Yanglin is characterized by steep slopes and rugged terrain, through which the river flows in narrow valleys. As it continues towards Xingping, the river meanders through a broader floodplain with gentle slopes. In the areas of Xianyang and Xi'an, the basin expands further, and the river forms a large alluvial plain with fertile soils. Finally, the lower reaches of Weinan exhibit a well-developed floodplain.

In the sampling area, the sediments in the interaction zone consist of silt, coarse sand, and clay, which are arranged in alternating layers. For the purpose of this study, soil was chosen as the experimental medium due to its typicality and ease of sampling. The sediment samples collected from the HZ in the sampling area consisted of layers alternating between silt, coarse sand, and clay. The sampling procedure followed the methodology described in a previous study^31^. The collected soil samples were carefully placed on clean, dry geotextile to allow for natural air drying. Once the soil was fully dried, it was sieved using a 1 mm diameter sieve to remove any tree roots, grassroots, and other impurities. Subsequently, experimental soil samples were obtained by sieving the soil through sieves with diameters of 0.1 mm, 0.25 mm, and 0.5 mm to obtain fine sand (0.10 mm < diameter < 0.25 mm), medium sand (0.25 mm < diameter < 0.5 mm), and coarse sand (diameter > 0.5 mm) fractions, respectively.

Laboratory ultrapure water was used as the practical water for the experiments. The composition of the soil particles and their corresponding parameters are presented in Table 1.

**Table 1:** Soil particle composition and parameters of the soil used.

Sampling point	Soil type	Particle size/mm	pH	Organic matter content (g/kg^−1^)
Column A	Coarse sand	0.5–1.0	7.87	1.112
Column B	Medium sand	0.20–0.5	7.72	0.654
Column C	Fine sand	0.120–0.20	7.25	0.265

### Experimental device

The experiment utilized an organic glass column with an inner diameter of 12 cm and a height of 70 cm. The column had an opening at the top and was connected at the bottom to a pressure-measuring tube and a Markov bottle through a three-way valve. A peristaltic pump was positioned between the Markov bottle and the column to regulate the water level and simulate groundwater fluctuation (rise and drop). The pressure-measuring tube was securely fixed on the soil column.

**Figure 1:**
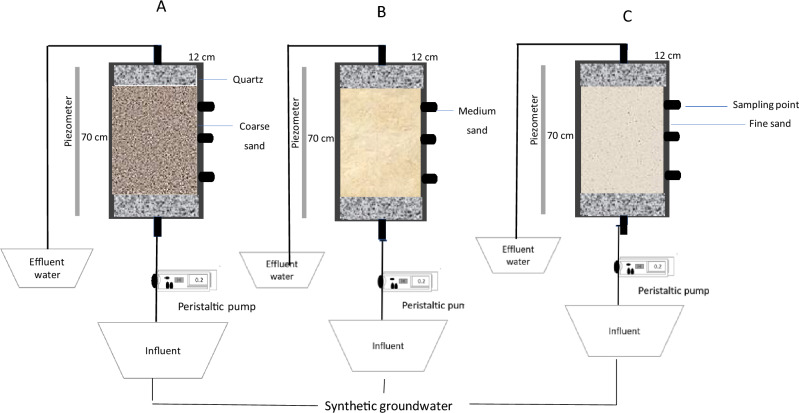
Schematic diagram of the experimental setup.

The organic glass column had five sampling points from top to bottom, but only three points were used for this experiment. The sampling points were located at distances of 10 cm, 30 cm, and 50 cm from the bottom of the column, with adjacent point intervals of 10 cm, 20 cm, and 20 cm, respectively. The Rhizon SMS (Single Matrix Sampler) was employed at each sampling point. The manufacturer of the Rhizon SMS was Shanghai Saifu Bio-Tech Co., LTD. in Shanghai, China. The top and bottom of each column were covered with a layer of 5 cm quartz sand (diameter 2 ~ 3 mm) to maintain a constant rise and fall of the water level. The experimental device is depicted in Fig. 1.

### Experimental design

The soil column experiment device, illustrated in Fig. 1, consisted of three pillars filled with the same quality of soil for different mediums. Each medium was filled to a height of 10 cm and compacted with a certain degree of strength. The soil medium was packed to the desired level, and the filling process was repeated until the column contained approximately 55 cm of soil medium. The soil solution sampler was placed at depths of 10 cm, 30 cm, and 50 cm within the column.

The upper opening of the column was sealed with plastic wrap to prevent moisture evaporation and later removed at the beginning of sample collection. Distilled water was introduced into the column from the Markov bottle using a peristaltic pump, stabilizing the water level at a 25 cm scale. The initial water level was maintained for one week to allow for capillary effects within the soil column and to establish a new environment. A small pipe connected to the air was attached to the mouth of the Markov bottle. The water level was controlled by observing the pressure measuring tube on the side of the soil column.

For the experiment, a 1 L solution of potassium nitrate with a concentration of 300 mg/L and a 1 L solution of ammonium chloride with a concentration of 300 mg/L were prepared. The solutions were uniformly poured onto the top of the soil column, and the water level was adjusted using the peristaltic pump to stabilize the internal water at 25 cm for 1 week. After 1 week, water samples were collected at each sampling point to determine the initial N concentrations.

The water level underwent periodic changes, with a 1-week stabilization period following each change. The first periodic change occurred after twelve days, followed by changes after twenty-seven and thirty-one days, completing a total of three periods. The flow rate of the inlet and outlet water was controlled at 100–200 mL min^−1^. At each water level change, the soil solution sampler was connected using a diaphragm vacuum pump. Water samples were collected at the four sampling points, and measurements were taken for pH, temperature, dissolved oxygen, NH_4_^+^-N, NO_3_^−^-N, and NO_2_^−^-N concentrations at each sampling point.

To determine the pH value, a pH meter (Model: Micro600; Manufacturer: Palintest Co., Ltd., Beijing, China) was utilized. The temperature was measured using a thermometer. Dissolved oxygen was measured using a dissolved oxygen meter (Model: JPBJ-608; Manufacturer: Shanghai Yifen Scientific Instrument Co., Ltd., Shanghai, China). And the concentrations of NO_3_^−^-N, NO_2_^−^-N, and NH_4_^+^-N were determined using Flow Injection Analyzer (FIA) (San++skaler)Shanghai Yifen Scientific Instrument Co., Ltd., Shanghai, China).

### Statistical analysis

In this research on the implications of water levels and soil texture on N migration and transformation in soil columns, Descriptive statistics were used to calculate the mean, standard deviation, and range of N concentrations at different soil depths and water levels, revealing overall patterns and variations. Analysis of Variance (ANOVA) was conducted to assess the statistical significance of differences in N concentrations among different water levels and soil textures.

## Results

### Physicochemical parameters in the effluents

**Figure 2:**
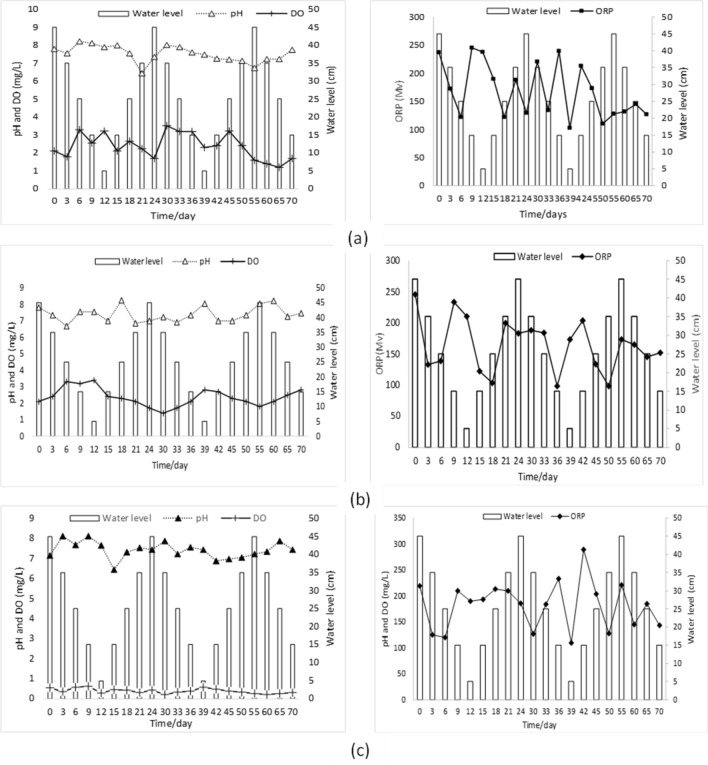
Changes in pH and DO and ORP in Different Soil types under varying water levels. The figures show the data collected from three columns containing coarse soil (**a**), medium sand (**b**) and fine sand (**c**), and the water level was varied from 45 to 5 cm in three cycles. Samples were collected every three days for the first two cycles and every five days for the last cycle.

The study spanned a duration of 70 days and aimed to investigate the variations in temperature, pH, ORP, and DO content in different soil types (coarse sand, medium sand, and fine sand) under varying water levels. As depicted in Fig. 2a in the coarse sand soil column, the water level fluctuated between 5 and 45 cm throughout the experiment. But no clear trend observed in pH values ranged from 6.44 to 8.22. The DO levels varied between 1.2 and 3.5 mg/L, reaching a minimum of 1.2 mg/L on the 65th day. However, ORP values (103 mV to 245 mV) shows fluctuations throughout the experiment.

In the medium sand soil column (Fig. 2b), the pH values varied between 6.66 and 8.23. The DO levels fluctuated between 1.4 and 3.5 mg/L, with a peak of 3.4 mg/L on the 12th day. The pH and DO levels showed both decreasing and increasing trends over time while for the fine sand soil column (Fig. 2c), the pH values ranged from 6.44 to 8.12, displaying an increasing trend in the early stages and later fluctuating. The DO levels varied between 1.2 and 3.5 mg/L, with both increasing and decreasing trends observed and ORP values ranged from 110 to 289 mV, exhibiting fluctuations without a consistent pattern. Analysis of varience (ANOVA) confirmed the variation in physicochemical properties observed across the soil columns were statistically significant (p < 0.05). These findings demonstrate the dynamic nature of the physicochemical parameters in the different soil types, influenced by changes in water level over time. The pH, dissolved oxygen, and ORP exhibited varying patterns, indicating the complex interplay between the water level and the soil's chemical environment. These results provide valuable insights into the characteristics of the soil and its potential impact on various processes within the hyporheic zone.

### Analysis of N migration and transformation

#### Variation of NO_3_^−^-N change law

**Figure 3:**
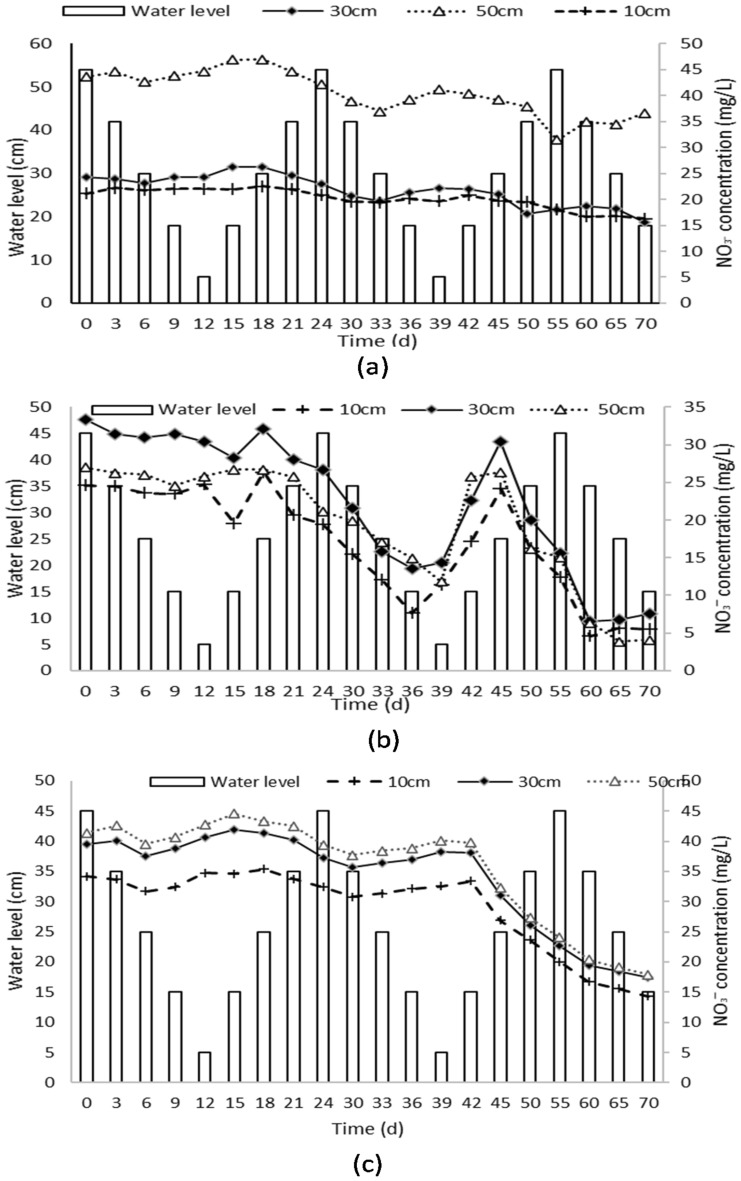
Changes in NO_3_^−^-N concentrations in different soil types under varying water levels. The figures show the data collected from three columns containing coarse soil (**a**), medium sand (**b**) and fine sand (**c**), and the water level was varied from 45 to 5 cm in three cycles. Samples were collected every three days for the first two cycles and every five days for the last cycle.

This study investigate the migration and transformation of NO_3_^−^-N within a soil column under different water levels and environmental conditions. To understand the movement and changes of NO_3_^−^-N, concentrations were monitored over a 70-day period at three distances within the soil column (10 cm, 30 cm, and 50 cm). The relationship between water levels and NO_3_¯-N concentrations is shown in Fig. 3a–c.

In Column A (Coarse sand) as shown in Fig. 3a, the initial mean concentration of NO_3_^−^-N at the 10 cm distance was 34.19 ± 0.86 mg/L on day 0 and gradually decreased to 14.33 ± 0.77 mg/L on day 70. Similar decreasing trends were observed at the 35 cm and 45 cm distances, indicating the migration or transformation of NO_3_^−^-N within the column. At the 30 cm sampling point, fluctuations in NO_3_^−^-N concentration were observed with changing water levels. For instance, on day 0, with a water level of 45 cm, the concentration was 29.22 ± 0.75 mg/L. However, on day 15, with a lower water level of 15 cm, the concentration increased to 31.53 ± 1.08 mg/L. Subsequently, on day 30, with a higher water level of 35 cm, the concentration decreased to 24.89 ± 0.77 mg/L. Similar patterns were observed at the 50 cm distance, indicating the influence of water levels on NO_3_^−^-N concentrations.

In Column B (Medium sand), the concentration of NO_3_^−^-N at the 10 cm distance started at 24.61 ± 0.59 mg/L on day 0, decreased to 7.65 ± 1.20 mg/L on day 36, and then slightly increased to 11.45 ± 1.45 mg/L on day 39, and further to 17.18 ± 0.99 mg/L on day 42. After day 42, the concentration fluctuated between 5.52 ± 1.05 mg/L and 17.18 mg/L due to water level fluctuations. Similar decreasing trends were observed at the 35 cm and 45 cm distances. Fluctuations in NO_3_^−^-N concentration were also observed at the 30 cm and 50 cm distances, corresponding to changes in water levels.

In Column C (Fine sand), the concentration of NO_3_^−^-N at the 10 cm distance started at 21.14 ± 0.24 mg/L on day 0 and fluctuated over time, reaching a peak of 22.20 ± 0.29 mg/L on day 3 and gradually decreasing to a minimum value of 16.29 ± 0.45 mg/L on day 70. Similar patterns of decreasing concentrations were observed at the 30 cm and 50 cm distances. Fluctuations in NO_3_^−^-N concentration were observed at the 30 cm and 50 cm distances, corresponding to changes in water levels.

The main theme highlighted in these findings is the variation of NO_3_^−^-N concentrations within the soil column under different water levels and distances. Both columns showed decreasing trends in NO_3_^−^-N concentrations over time, with fluctuations corresponding to changes in water levels. This indicates the migration or transformation of NO_3_^−^-N within the soil column and highlights the complex relationship between water levels and NO_3_^−^-N concentrations.

#### Variation of NH_4_^+^-N change law

The concentration of NH_4_^+^-N in different sand columns (coarse sand, medium sand) was monitored over a 70-day period to understand the relationship between water levels and NH_4_^+^-N concentrations. The following observations were made: Coarse sand column (Column A): At a sampling point located 10 cm below the water level, the initial NH_4_^+^-N N concentration on day 0 was 3.69 ± 0.30 mg/L, with a water level of 45 cm. Surprisingly, on day 15, despite the water level dropping to 15 cm, the concentration increased to 6.88 ± 0.38 mg/L. On day 30, with a higher water level of 35 cm, the concentration decreased to 3.77 ± 0.23 mg/L. The lowest NH_4_^+^-N concentration was observed on day 55, with a water level of 45 cm, measuring 2.69 ± 0.17 mg/L. Finally, on day 70, with a water level of 15 cm, the NH_4_^+^-N concentration reached its lowest recorded value of 1.51 ± 0.13 mg/L. Similar trends were observed at sampling points 30 cm and 50 cm below the water level, suggesting a relationship between water levels and NH4^+^-N concentrations in the coarse sand column.

Medium sand column (Column B): Fluctuations in NH_4_^+^-N concentrations were observed corresponding to different water levels over the 70-day period. At a sampling point 10 cm below the water level on day 0, with a water level of 45 cm, the NH_4_^+^-N concentration was 2.74 ± 0.43 mg/L. Surprisingly, on day 15, with a lower water level of 15 cm, the concentration increased to 3.90 ± 0.30 mg/L. On day 30, with a water level of 35 cm, the concentration decreased to 2.59 ± 0.26 mg/L. The lowest NH_4_^+^-N concentration was observed on day 70, with a water level of 15 cm, measuring 1.39 ± 0.30 mg/L. Similar patterns were observed at sampling points 30 cm and 50 cm below the water level, indicating variability in NH_4_^+^-N concentrations in response to different water levels in the medium sand column.

Fine sand column (Column C): NH_4_^+^-N concentrations were monitored at different sampling points and water levels over a 70-day period. At a sampling point located 10 cm below the water level on day 0, with a water level of 45 cm, the average NH_4_^+^-N concentration measured was 9.260 ± 0.06 mg/L on day 15, despite the water level being 15 cm lower, the mean NH_4_^+^-N concentration dropped to 11.660 ± 0.03 mg/L. Further observations showed a decrease in NH_4_^+^-N concentration to 6.790 ± 0.23 mg/L on day 30, with a water level of 35 cm. On day 55, with a water level of 45 cm, the NH4 + -N concentration decreased even further to 3.690 ± 0.13 mg/L. Finally, on day 70, with a water level of 35 cm, the NH_4_^+^-N concentration dropped significantly to 0.670 ± 0.20 mg/L. Similar trends were observed at a sampling point located 30 cm below the water level.

Transformation of NH_4_^+^-N NH_4_^+^-N into NO_3_^−^-N (nitrification): The mean values of NH_4_^+^-N at a depth of 50 cm showed distinct patterns corresponding to different water levels. On day 0, with a water level of 45 cm, the NH_4_^+^-N concentration was 0.074 ± 0.02 mg/L. On day 15, with a lower water level of 15 cm, the concentration increased significantly to 4.08 ± 0.14 mg/L. On day 30, with a water level of 35 cm, the concentration decreased to 0.38 ± 0.54 mg/L. On day 55, with a water level of 45 cm, the NH_4_^+^-N concentration increased to 0.89 ± 0.05 mg/L. Finally, on day 70, with a water level of 15 cm, the concentration decreased to 0.55 ± 0.27 mg/L.

Overall, the NH_4_^+^-N concentrations showed a decreasing trend throughout the experiment at each sampling point. However, the dynamics of NH_4_^+^-N were influenced by variations in the water level. As the water level rose, the NH_4_^+^-N concentration increased, and when the water level dropped, the NH_4_^+^-N concentration decreased. This trend of NH_4_^+^-N concentration. The trend of NH_4_^+^-N concentration showed an opposite pattern compared to the observed fluctuations in NO_3_^−^-N concentration in response to changes in water level.

**Figure 4:**
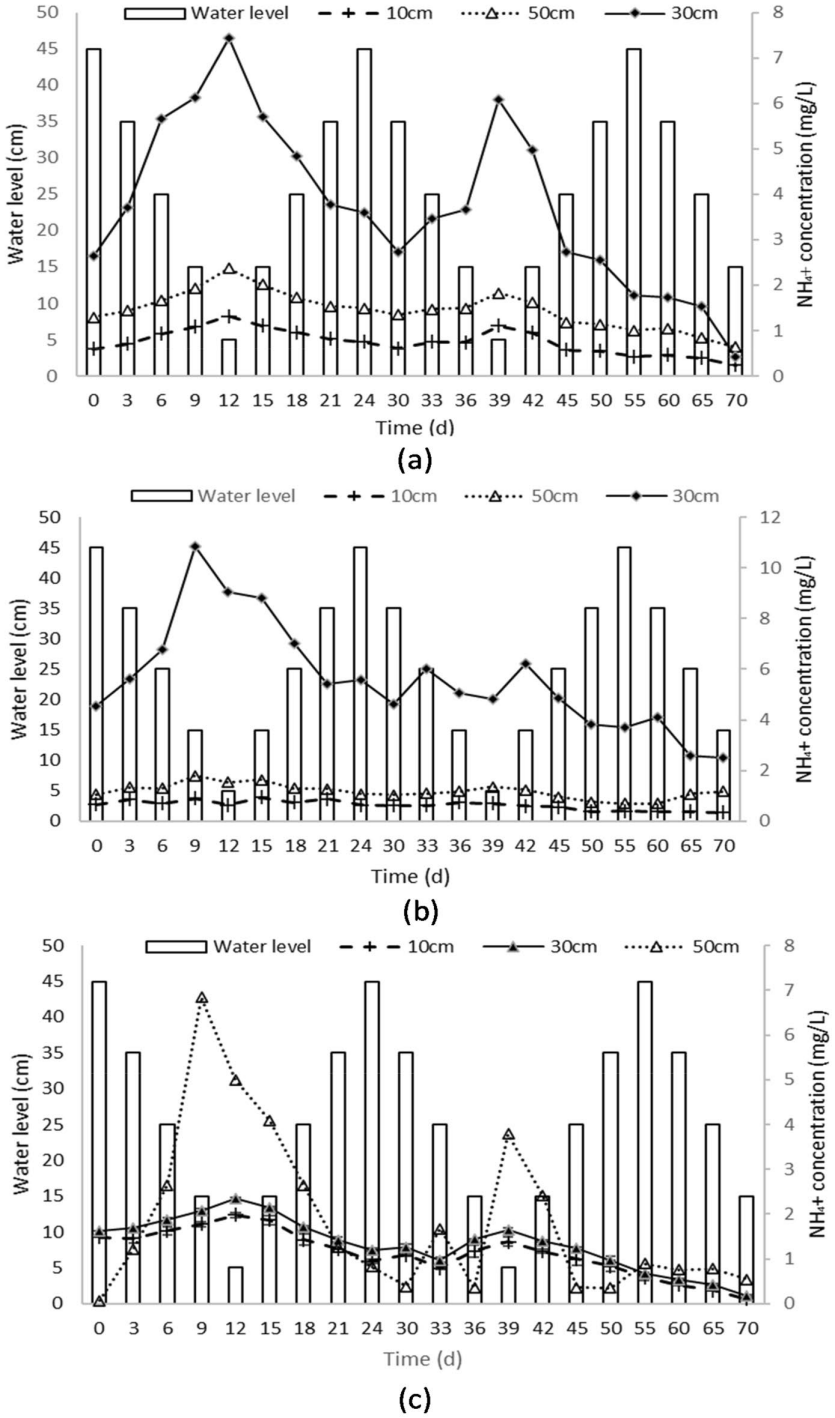
Changes in NH_4_^**+**^-Concentrations in different soil types under varying water levels. The figures show the data collected from three columns containing coarse soil (**a**), medium sand (**b**) and fine sand (**c**), and the water level was varied from 45 to 5 cm in three cycles. Samples were collected every three days for the first two cycles and every five days for the last cycle.

**Figure 5:**
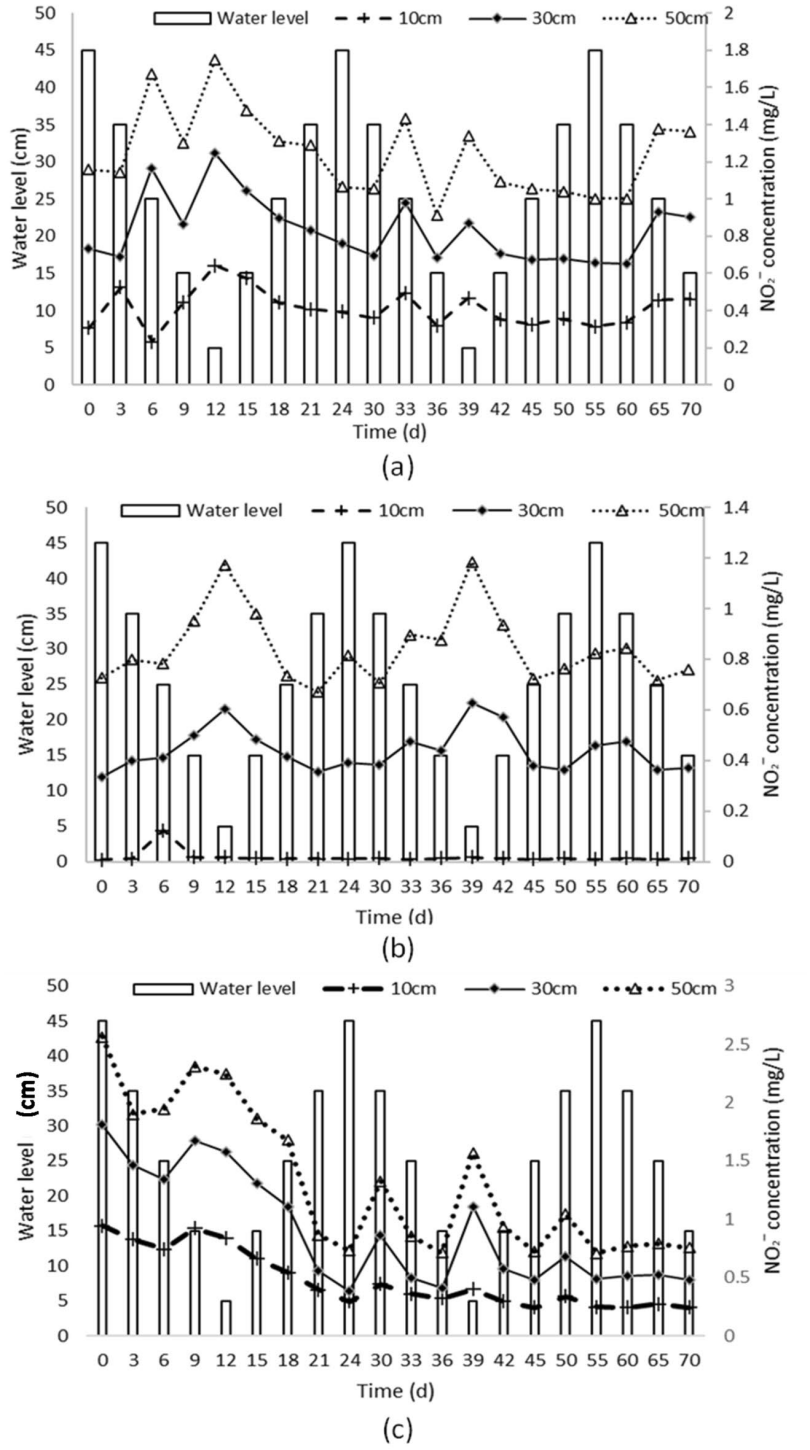
Changes in NO_2_^−^—Concentrations in different soil types under varying water levels. The figures show the data collected from three columns containing coarse soil (**a**), medium sand (**b**) and fine sand (**c**), and the water level was varied from 45 to 5 cm in three cycles. Samples were collected every three days for the first two cycles and every five days for the last cycle.

Comparing Figs. 3, 4, and 5, it is evident that the concentration of NO_2_^−^-N is much lower compared to nitrate nitrogen (NO_3_^−^-N) and NH_4_^+^-N.

Column A (Coarse sand): Fig. 5a shows that the migration and transformation of NO_2_^−^-N in Column A, which was filled with coarse sand, were influenced by fluctuations in the water level. The water level in Column A fluctuated between 35 and 45 cm during the experiment, indicating changes in hydraulic conditions and nutrient transport within the column. NO_2_^−^-N concentrations varied spatially and temporally at different depths (10 cm, 30 cm, and 50 cm). The concentrations differed among the depths at each time point, emphasizing the importance of considering depth-dependent variations when analyzing NO_2_^−^-N migration and transformation Temporal variations in NO_2_^−^-N concentrations at each depth suggest dynamic processes of nitrogen transformation and transport influenced by factors like microbial activity, oxygen availability, and substrate availability.

Column B (Medium sand): Fig. 5b focuses on Column B, which contained medium sand. It shows that the migration and transformation of NO_2_¯-N in this column were influenced by water level fluctuations. The water level in Column B also fluctuated between 35 and 45 cm throughout the experiment. NO_2_^−^-N concentrations varied spatially and temporally at different depths (10 cm, 30 cm, and 50 cm), indicating dynamic nitrogen transformation and transport within the column. The depth-dependent variations highlight different NO_2_^−^-N concentrations at each depth at each time point, while the time-dependent variations demonstrate changes in NO_2_^−^-N concentrations over the course of the experiment.

Column C (Fine sand): The analysis of Column C, filled with fine sand, showed variations in NO_2_^−^-N concentrations at different depths and over time during the 70-day experiment. Initial concentrations at depths of 10 cm, 30 cm, and 50 cm were 0.94 mg/L, 0.87 mg/L, and 0.76 mg/L, respectively. Towards the end of the experiment, these concentrations decreased to 0.24 mg/L, 0.27 mg/L, and 0.25 mg/L at the same depths. Water level fluctuations were found to influence the migration and transformation of NO_2_^−^-N in the fine sand environment. Comparisons with other soil columns indicated that Column C had a moderate absorption capacity for NO_2_^−^-N, with concentrations higher than in Column A (coarse sand) but lower than in Column B (medium sand). The absorption capacity order for NO_2_^−^-N, from highest to lowest, was Column B > Column C > Column A.

## Discussion

### Variation characteristics of physicochemical parameters in the experimental effluent

This study provides valuable insights into the variations of physicochemical parameters in the experimental effluent, including water level, pH, dissolved oxygen (DO), and oxidation–reduction potential (ORP). These variations are closely related to the migration and transformation of nitrogen (NO_3_^−^-N) within different sand types (coarse sand, medium sand, and fine sand) in the soil columns. Understanding these variations is crucial for comprehending the complex dynamics of nitrogen movement and its environmental implications. In the coarse sand soil column, the water level exhibited fluctuations throughout the study, which can significantly impact the movement of nitrogen within the soil. These fluctuations affect the saturation and aeration of the soil, which in turn influence the availability of oxygen and the processes of nitrification and denitrification^13,14,32^. These processes, in turn, affect the concentrations of NO_3_^−^-N in the effluent. The pH values in the coarse sand soil showed similar fluctuations to previous studies, indicating the influence of microbial activity and nutrient availability. The activities of soil microorganisms involved in nitrogen cycling, such as nitrifying and denitrifying bacteria, are known to be influenced by pH levels^7^. Therefore, the variations in pH can indirectly affect the migration and transformation of NO_3_^−^-N within the soil column. The DO levels in the coarse sand soil displayed variations comparable to the medium and fine sand soils, reflecting changes in oxygen availability^33^. The presence of oxygen is crucial for the activity of nitrifying bacteria, which convert ammonium (NH_4_^+^) into nitrate (NO_3_^−^), and the absence of oxygen promotes denitrification, leading to the loss of NO_3_^−^-N^3^. The fluctuations in ORP in the coarse sand soil, similar to the medium sand soil, suggest variations in the redox reactions occurring in the soil^8^. Redox conditions play a significant role in determining the availability of electron acceptors and donors, affecting the transformation of nitrogen compounds.

In the medium sand soil column, the variations in pH, DO, and water level also contribute to the migration and transformation of NO_3_^−^-N. Fluctuations in pH, influenced by factors such as microbial activity and nutrient availability, can affect the activities of nitrifying and denitrifying bacteria, leading to changes in NO_3_^−^-N concentrations^32^. Additionally, changes in DO levels, influenced by microbial respiration, oxygen diffusion, and oxygen consumption by plants and organisms, can affect the rates of nitrification and denitrification, thereby influencing NO_3_^−^-N concentrations^23^. The fluctuations in water level impact the availability of water and soil moisture, which can influence microbial activity and the movement of NO_3_^−^-N within the soil.

In the fine sand soil column, variations in pH, DO, and ORP also play a crucial role in the migration and transformation of NO_3_^−^-N. Changes in pH, influenced by factors such as acidic or alkaline compounds, microbial activity, and nutrient availability, can affect the activity of nitrifying and denitrifying bacteria, influencing the concentrations of NO_3_^−^-N. Fluctuations in DO levels, influenced by microbial respiration, organic matter decomposition, and oxygen diffusion, can impact the rates of nitrification and denitrification^7^. The variations in ORP reflect changes in the availability of electron acceptors and donors, affecting the redox reactions involved in nitrogen transformation.

Therefore, the variations in physicochemical parameters observed in the experimental effluent are closely related to the migration and transformation of NO_3_^−^-N within the soil columns of different sand types. Factors such as water level, pH, DO, and ORP influence the activities of microorganisms involved in nitrogen cycling, affecting the concentrations of NO_3_^−^-N^10^. Understanding these variations and their relationships with nitrogen dynamics is crucial for managing nutrient pollution, assessing soil health, and promoting sustainable agricultural practices^32^. Further research is needed to explore the specific mechanisms linking physicochemical parameters with nitrogen migration and transformation, allowing for more effective strategies for nitrogen management in agricultural and environmental contexts^17^.

### Variation characteristics of inorganic N concentrations in the experimental effluent

The variation characteristics of inorganic nitrogen (N) concentrations in the experimental effluent provide valuable insights into the migration and transformation of NO_3_^−^-N, NH_4_^+^-N, and NO_2_^−^-N in different soil types. Fluctuations in concentration can be attributed to factors such as microbial activity, water flow, porosity, and transformations occurring within the column^26–29^. Soil texture plays a significant role in the movement and retention of water and nutrients, including NO_3_^−^-N. Medium sand, with its balanced composition of sand, silt, and clay particles, provides moderate water-holding capacity and nutrient retention. This allows water and NO_3_^−^-N to move more freely through the soil profile. Fine sand, with the largest particle sizes, results in faster drainage and reduced water and nutrient retention. These differences in soil texture affect the availability and movement of NO_3_^−^-N within the soil^10^. The sampling port above the water level showed no significant changes and was less affected by water level fluctuations. However, the sampling port below the water level was significantly affected by water level fluctuations, with the concentration of NO_3_^−^-N showing a significant decreasing trend during the water level fall stage. During the rising stage of water level fluctuation, the NO_3_^−^-N content in the soil solution greatly reduced, while during the dropping stage, the corresponding content significantly increased. This can be attributed to the decrease in dissolved oxygen content during the rising stage and the increase in dissolved oxygen concentration during the falling period^34^. The nitrification reaction is influenced by dissolved oxygen concentration, and a decrease in NO_3_^−^-N content leads to the accumulation of NH_4_^+^-N. The migration and transformation of NO_3_^−^-N in soil are influenced by factors such as soil water content, water flow movement state, dissolved oxygen content, and microbial community. Fluctuations in water level cause significant changes in soil water content, water flow movement state, and dissolved oxygen content in the soil section below the water level, leading to notable variations in NO_3_^−^-N content in the soil solution in that particular section.

In the coarse sand soil, there was a rapid downward migration of NO_3_^−^-N above the water level due to its weak adsorption capacity for NO_3_^−^-N. Additionally, a strong denitrification effect above the water level resulted in low concentrations of NO_3_^−^-N. When the water level rose, there was a significant upward movement of NO_3_^−^-N below the water level, as the physical application caused by the rising water level outweighed the denitrification effect. Conversely, in fine sand, which has a strong adsorption capacity for nitrate N, there was minimal downward migration of NO_3_^−^-N above the water level in response to groundwater level fluctuations. The adsorption capacity of fine-sand soil hindered the migration of NO_3_^−^-N.

During the rising stage of water level fluctuations, there was a significant increase in NH_4_^+^ content in the soil solution. However, during the falling stage, there was a noticeable decline in NH_4_^+^-N concentration. The measured pH values and dissolved oxygen levels support the finding that lower dissolved oxygen concentrations contribute to higher NH_4_^+^-N concentrations in the soil solution^35^. Conversely, higher dissolved oxygen concentrations significantly reduce NH_4_^+^-N concentrations. This pattern can be explained by the rise in water level, which leads to a decrease in dissolved oxygen content and a transition from an aerobic stage to an anoxic stage. Denitrifying bacteria become more active, enhancing their ability to reduce compounds, leading to an increase in NH_4_^+^-N concentration and a decrease in NO_3_^−^-N concentration. Conversely, as the water level decreased, the dissolved oxygen content increased, promoting oxidation and the nitrification reaction. The effect of nitrification outweighed denitrification, leading to an increase in the mass concentration of NO_3_^−^-N. Furthermore, the soil's strong adsorption capacity for NH_4_^+^-N limits its accumulation in soil-free water, contributing to an overall downward trend in NH_4_^+^ concentration. These findings align with previous research on variations in NH_4_^+^-N and NO_3_^−^-N concentrations in response to water level fluctuations. The fluctuations in NO_2_^−^-N concentration over time can be attributed to various factors, including microbial activity, water flow, and transformations occurring within the soil column. The concentrations of NO_2_^−^-N at different sampling points were influenced by the fluctuating water levels within the column. In general, the concentrations of nitrate N decreased over the course of the study, suggesting migration or transformation away from the sampling points. At the sampling points below the water level, the concentration of NO_2_^−^-N showed a significant decreasing trend during the rising stage of water level fluctuation. This may be attributed to a decrease in dissolved oxygen content during the rising stage, which impacts the nitrification reaction. In contrast, during the falling stage of water level fluctuation, there is an increase in dissolved oxygen concentration, resulting in an increase in nitrate N concentration. The rate of nitrification is accelerated as the dissolved oxygen concentration rises.

Overall, these findings imply that water level fluctuations can impact the migration and transformation of NO_2_^−^-N in a column filled with coarse sand. The fluctuating water levels likely influence the transport pathways, residence times, and interactions with the microbial community in the sediment. During the water level rise phase, there is an accumulation of NO_2_^−^-N, which could be attributed to the lower growth rate of nitrifying bacteria compared to denitrifying bacteria when dissolved oxygen is fully saturated. This leads to a higher rate of nitrification than denitrification, resulting in an increase in NO_2_^−^-N concentration. However, when the water level rises, the decrease in dissolved oxygen content affects the nitrification reaction^3^. In the water level decrease phase, the increase in dissolved oxygen concentration in the soil profile enhances the nitrification process, causing the nitrification reaction to produce H^+^ ions. Over time, this leads to a weakly acidic solution. Studies have shown that pH values have a significant negative correlation with nitrate content but no significant correlation with ammonium and nitrite^33,36,37^. Therefore, a weakly acidic solution promotes the conversion of nitrite to nitrate, but it has no significant effect on ammonium and nitrite, resulting in no accumulation of nitrite and a gradual decrease in its content. Consequently, the findings demonstrate that the NO_2_^−^-N content fluctuates continuously throughout the experiment and eventually reaches a stable with obervable patterns.

The observed differences in absorption capacities among the soil columns can be attributed to variations in soil texture, porosity, and permeability. The medium sand in Column B creates more favorable conditions for NO_2_^−^-N absorption, while the coarse sand in Column A may have limited capacity due to its coarse texture. The fine sand in Column C falls in between, exhibiting intermediate absorption capabilities. These findings emphasize the importance of soil characteristics in determining the absorption capacity of NO_2_^−^-N. Understanding the variations in absorption capacities among different soil types is crucial for managing and mitigating N pollution and can inform agricultural and environmental practices to optimize nutrient retention in soil systems^10,11,31^.

The migration and transformation of NO_2_^−^-N in soil are influenced by factors such as soil water content, water flow movement state, dissolved oxygen content, and microbial community^33,35,38–40^. The observed changes in soil water content, water flow movement state, and dissolved oxygen content below the water level due to fluctuations in the water level were found to be the main drivers behind the variations in nitrate N content in the soil solution in that particular section. It is important to note that the dynamics of nitrate–N migration and conversion can vary depending on the soil type. Coarse sand soil exhibited rapid downward migration of NO_2_^−^-N, while fine sand soil, with its strong adsorption capacity, hindered the migration of nitrate N above the water level in response to groundwater level fluctuations. Overall, this study highlights the complex relationship between water levels, soil characteristics, and N concentrations.

## Conclusion

The concentrations of NO_3_^−^-N within the soil column exhibited fluctuations and migration influenced by water levels and soil texture. Higher water levels were associated with decreased nitrate N concentrations, while lower water levels resulted in increased concentrations. Fine sand soil demonstrated the highest retention and absorption capacity for nitrate N, followed by medium sand and coarse sand, emphasizing the significant role of soil texture in nitrate movement and retention. Variations patterns were observed in NO_3_^−^-N concentrations within the soil column. Fluctuating water levels influenced the migration and transformation of NO_3_^−^-N, with distinct patterns observed in coarse sand, medium sand, and fine sand. Water level fluctuations also had a significant impact on the migration and transformation of NH_4_^+^-N, with higher water levels associated with increased concentrations and lower water levels leading to decreased concentrations. Soil type influenced the absorption capacity of NH_4_^+^-N, with medium sand exhibiting the highest absorption capacity. Overall, the research findings highlight the crucial roles of water levels, soil texture, and soil type in the migration, transformation, and absorption of N compounds within soil columns. These conclusions align with the research objectives of understanding the dynamics of N migration and transformation under varying water levels and environmental conditions.

## Data Availability

The datasets generated and/or analysed during the current study are not publicly available In order to protect the privacy of my research data and that of the researchers involved, the original data will not be provided. The data are presented in various figures and tables in the manuscript but are available from the corresponding author on reasonable request.
